# Infections and systemic lupus erythematosus

**DOI:** 10.1590/S1679-45082016AO3490

**Published:** 2016

**Authors:** Thelma Larocca Skare, Jéssica Scherer Dagostini, Patricia Imai Zanardi, Renato Mitsunori Nisihara

**Affiliations:** 1 Faculdade Evangélica do Paraná, Curitiba, PR, Brazil.; 2 Universidade Positivo, Curitiba, PR, Brazil.

**Keywords:** Lupus eryithematosus, systemic/complications, Urinary tract infection/etiology, Immunosuppression

## Abstract

**Objective:**

To determine the incidence of infections in a population of systemic lupus erythematosus individuals and the characteristics of infections regarding original site, as well as to study the possible associations between infections and treatment.

**Methods:**

An analytical retrospective study using data from medical charts of systemic lupus erythematosus patients from a single university hospital. A total of 144 patients followed up for five years were included. Data collected comprised age of patients and age at onset of lupus, sex and ethnicity, disease duration before the study period, medications, cumulative dose of prednisone, occurrence of infections and their original site.

**Results:**

The most frequent infections were urinary tract infections (correlated to use of prednisone − p<0.0001 and cyclophosphamide − p=0.045), upper airways infections (correlated to use of prednisone − p=0.0004, mycophenolate mofetil − p=0.0005, and cyclosporine − p=0.025), and pneumonia (associated to prednisone − p=0.017).

**Conclusion:**

Prednisone was the drug more often associated with presence of infections, pointing to the need for a more judicious management of this drug.

## INTRODUCTION

Systemic lupus erythematosus (SLE) is an multisystem chronic inflammatory disease, with autoimmune characteristics and unknown etiology, related to genetic, environmental, and hormonal factors.^1^ It predominantly affects women during child-bearing age and tends to be more common and more severe in black and Asian individuals.^1^

Systemic lupus erythematosus is a potentially severe disease.^1^ Since it is a type of vasculitis of the medium-sized and small vessels, all organs are subject to damage, including those whose function is vital for survival, such as heart, lungs, kidneys, and central nervous system.^1^ Nevertheless, with new armamentarium for therapy and making early diagnosis, the acute flares of the disease can be better controlled; mortality due to vasculitis has diminished and the disease took on a more chronic nature.^2^

In 1976, Urowitz et al.^3^ observed a bimodal pattern in causes of death in SLE: an initial peak, due to activity of the disease and infections, and another late peak caused by coronary artery disease and infections. In the subsequent years, several other authors confirmed these patterns, which have become known as the bimodal distribution of Urowitz.^2,3^ Infections are one of the main causes of morbidity and mortality in this disease, whether early or late, and all effort should be focused on their prevention and treatment.

Patients with SLE seem to have an increased risk for infections. Among the defects of the immune system, there is a decrease in T CD4+ lymphocytes, deficiency of the complement system components, neutropenia, and lymphopenia.^4-6^ Some authors described the activity of the disease as a risk factor for infections, especially when it involves certain organs, such as the kidneys, and/or central nervous system.^5,7,8^

The risk of infections in SLE is greater due to the use of immunosuppressants and particularly glucocorticoids, widely used for treatment of the systemic complications of this disease.^9^ The latter medication does not yet have the desired specificity, which would be that of acting only on self-reactive and dysfunctional cells, and ends up weakening the normal defenses of the body and favoring the aggression of microorganisms.

## OBJECTIVE

To determine the incidence of infections in a population with systemic lupus erythematosus and its characterization as to original site, in addition to studying the possible associations of infections and treatment used.

## METHODS

This study has an analytic retrospective design and used data from medical records of patients with SLE, seen at the Rheumatology Service of the *HospitalEvangélico de Curitiba*, in the state of Paraná (PR). After approval by the Research Ethics Committee of the organization under number 32371614.6.0000.0103, a total of 144 patients diagnosed with SLE were investigated for a five-year period (December 1, 2007, to December 1, 2012). Patients of both sexes were studied, who presented with at least four of the classifying criteria for SLE as per those adopted by the American College of Rheumatology (ACR), in 1997.^10^ Excluded were patients aged under 16 years, individuals with HIV or forms of agammaglobulinemia.

The data collected included age of patient and age at onset of SLE; sex and ethnicity; duration of the disease before inclusion in the study; medications used; cumulative dose of prednisone administered; clinical profile (evaluated cumulatively and using a definition according to the ACR criteria);^10^ and serological profile, occurrence of infections, and their site. The infections were expressed in number of infections for every 100 patients/year.

In the statistical analysis, the central tendency measures were presented as means and standard deviation, for samples with Gaussian distribution, and medians and interquartile range (IQR) for non-Gaussian samples. For the study of frequency of symptoms, serology findings and use of medications, percentages were used. The correlation between presence of infections, medications, and epidemiological data was made using Spearman’s test. The significance level adopted was 5%. Calculations were made using the Graph Pad Prism statistical package, version 5.0.

## RESULTS

Among the patients studied, 135/144 (93.8%) were women, age ranged between 17 and 67 years (mean of 39.15±11.65 years) and the duration period of the disease before the patient being included in the present study was 9 to 483 months (median 87.0; IQR of 60.0-132.0 months).

The clinical and serological findings of the population studied, obtained in a cumulative manner during the course of the disease, showed that there was photosensitivity in 70.7%, malar erythema in 48.9%, oral ulcers in 45.6%, psychosis in 8.3%, convulsions in 11.1%, serositis in 20.1%, leukopenia in 27.8%, decreased platelet count in 22.3%, hemolytic anemia in 4.8%, glomerulonephrites in 42.3%, anti-dsDna in 33.3%, anti-Ro in 37.3%, anti-La in 21.2%, anti-Sm in 23.5%, and anti-RNP in 27.3%.

As to use of medications, 93.7% were on antimalarial drugs, 31.9% on cyclophosphamide, 17.3% on mycophenolate mofetil, 31.2% on methotrexate, 6.25% on thalidomide, 4.1% on cyclosporine, and 86.1% on prednisone. The cumulative dose of prednisone during the five-year observation varied from 0 to 56,758mg (median of 963mg; IQR of 2,491-18,647mg) and was calculated by multiplying the daily dose used by the number of days it was used.

In the sample studied, it was observed the occurrence of 1.68 urinary tract infection/100 patients per year; 1.56 upper airway infection/100 patients per year; 0.47 pneumonia/100 patients per year; 0.27 herpes zoster/100 patients per year; 0.27 candidiasis/100 patients per year; and 0.05 case tuberculosis/100 patients per year.

The study for correlation of the number of infections with the medications used is shown on table 1.

**Table 1:** Correlation between the use of medications and infections in patients with systemic lupus erythematosus*

Medication	Rho Spearman	95%CI	p value
Urinary tract infections			
Cyclophosphamide	0.16	0.001-0.32	0.04
Prednisone (CD)	0.34	0.18-0.48	<0.0001
Upper airway infections			
Mycophenolate mofetil	0.28	0.14-0.42	0.0005
Prednisone (CD)	0.28	0.12-0.43	0.0004
Cyclosporine	0.18	0.018-0.34	0.025
Pneumonia			
Prednisone (CD)	0.19	0.02-0.35	0.017
Herpes zoster			
Cyclophosphamide	0.31	0.15-0.45	0.0001
Prednisone (CD)	0.29	0.13-0.44	0.0003
Azathioprine	0.19	0.019-0.35	0.025

* The correlations of infections with medicines used by patients and not included in this table are not significant (p=NS); 95%CI = 95% confidence interval; CD: cumulative dose.

The presence of tuberculosis and bacterial skin infections were not significantly associated with the use of any kind of medication.

## DISCUSSION

Systemic lupus erythematosus can be a difficult to control disease.^1^ The objectives of its treatment are prevention of acute vasculitis flares, to avoid permanent lesion of the affected organs, maintenance of the disease in remission and/or delay its progression.^1^

Some medications, such as non-steroidal anti-inflammatory drugs and antimalarial agents are widely used as treatment. These are effective in the prevention of arthritis and skin manifestations, besides helping to keep the disease in remission in the case of the antimalarials.^1^

Glucocorticoids are used especially at the beginning of an acute flare of the disease due to their rapid action, and in doses that vary according to the degree and type of the organ involved.^1,10^ Despite more than 60 years of experience of their use, there are no guidelines on the ideal dosage of this medication in the different manifestations of the disease,^11^ and the doses are chosen as per clinical judgment of each situation. The rapid and potent action of the glucocorticoids often save the life of a patient; but its use causes a myriad of side effects, including facilitating the occurrence of infections.^11^ Sisó et al.^12^ consider glucocorticoids, along with cyclophosphamide, the drugs that most favor infections in this context. They have general inhibiting effects on T- and B-lymphocyte-mediated immune responses, as well as potent suppressive effects on the functions of monocytes and neutrophils, favoring opportunistic infections that are many times very severe, such as invasive pneumococcal disease.^13,14^The use of glucocorticoids has been associated with the appearance of respiratory and genitourinary tract infections caused by *Gram*-negative microorganisms,^4,15^ as well as infections caused by mycobacterial, cryptococci, listeria, and nocardia.^16^ A retrospective study with 2,717 SLE patients compared the population that used glucocorticoids (n=989) with one that did not use it (n=1,728). This study showed that the patients that used this medication at a mean daily dose of more than 7.5 mg/prednisone are more susceptible to the development of pneumonia, herpes zoster, and fungal infection.^17^ Other authors observed this same threshold of daily dosage can increase the possibility of urinary tract infections.^14,18^ Gladman et al.^19^ postulated that the continued use of corticoids – regardless of dose, duration, and route of administration, leads to a greater risk of infections. One alternative to solve this problem would be to adjust the dose of glucocorticoid for the type of cell predominantly responsible for the respective manifestation of SLE, knowing the effect of the glucocorticoids on different types of cells.^13^ Nonetheless, this knowledge about the pathophysiology of the disease, and even as to the mode of action of the glucocorticoids, is still far from being incorporated into daily practice.^13^ In the present study, the results found were consistent with those of literature, showing that the use of prednisone was associated with greater susceptibility to infections of the urinary tract, upper airways, and pneumonia.

Other drugs that make up the therapeutic armamentarium in SLE are immunosuppressants, such as methotrexate (indicated to treat some manifestations, such as myositis, arthritis, and skin lesions) and azathioprine (used in hematological manifestations and in maintenance of remission of nephritis).^1^ These two medications may be recommended as corticoid-sparing agents.^1^ Whereas cyclophosphamide and mycophenolate mofetil are used in more severe manifestations of the disease, such as involvement of the kidneys, nervous system, and larger vasculitis.^1^

Cyclophosphamide is a DNA alkylating agent that reduces the number of B- and T-lymphocytes, besides modulating the activation of these cells and the production of antibodies.^20^ Despite the controversy, some authors^7,21^ stated isolated therapy with cyclophosphamide does not seem to be a significant risk factor for infections, but that this risk is increased when the drug is combined with corticoids. In the literature, cyclophosphamide has been associated with urinary tract infections,^21-23^ corroborating the findings of the present study. The occurrence of hemorrhagic cystitis, one of the known complications of cyclophosphamide, may result in the appearance of urinary reflow, and this, on the other hand, favors more infections.^22,23^

A retrospective study on the use of mycophenolate mofetil in SLE showed that cystitis, upper airway infection, bronchitis, and cellulitis were the infections most frequently associated with this medication.^24^ In our series of cases, we noted an association between the use of this drug with upper airway infections.

In the present study, the infections by herpes zoster were associated with the use of cyclophosphamide, prednisone, azathioprine, and greater duration of disease. Infections by herpes zoster are due to the reactivation of the varicella-zoster virus, which could occur decades after initial exposure. Some authors described greater frequency of reactivation of this disease in lupus patients.^25,26^ Borba et al.^26^ reported 55 episodes of herpes zoster infections in 51 SLE patients, and the most noteworthy characteristic of this study was the fact that most episodes occurred five years after diagnosis of SLE, often appearing during periods of disease remission.

SLE is a complex disease. The progression of these patients is highly variable and requires cautious surveillance by the assisting physician. Although having a large impact on survival and quality of life of this population, the treatment may predispose towards repeated infections, which may eventually become severe. It is at the physician´s discretion to judiciously use these therapies, especially glucocorticoids, employing them for the shortest period and at the lowest dose possible.

## CONCLUSION

The most common infection in this sample of systemic lupus erythematosus patients was that of the urinary tract, followed by the upper airways. Urinary tract infections were significantly associated with the use of prednisone and cyclophosphamide; whereas respiratory tract infection, with the use of prednisone, mycophenolate mofetil and cyclosporine.

In general, glucocorticoid was the medication more often associated with the presence of infections, indicating the need for its adequate management.
