# Supplementary material for: Infections and systemic lupus erythematosus
Source: Einstein (Sao Paulo). 2016 Jan-Mar;14(1):47–51. doi: 10.1590/S1679-45082016AO3490 (PMC4872917; doi:10.1590/S1679-45082016AO3490)
Supplement: Translation

# Translation: Infecções e lúpus eritematoso sistêmico

**Authors:** Thelma Larocca Skare, Jéssica Scherer Dagostini, Patricia Imai Zanardi, Renato Mitsunori Nisihara

**Affiliations:** 1 Faculdade Evangélica do Paraná, Curitiba, PR, Brasil.; 2 Universidade Positivo, Curitiba, PR, Brasil.

**Keywords:** Lúpus eritematoso sistêmico/complicações, Infecções urinárias/etiologia, Imunossupressão

## Abstract

**Objetivo:**

Determinar a incidência de infecções em uma população com lúpus eritematoso sistêmico e a caracterização das mesmas quanto aos locais de origem, assim como estudar possíveis associações das infecções com a terapêutica utilizada.

**Métodos:**

Estudo retrospectivo analítico utilizando dados de prontuários de indivíduos com lúpus eritematoso sistêmico de um único hospital universitário. Foram investigados 144 pacientes acompanhados pelo período de 5 anos. Os dados coletados incluíam idade do paciente e idade de início do lúpus, sexo e etnia, tempo de duração da doença antes do período do estudo, medicamentos administrados, dose cumulativa de prednisona, ocorrência de infecções e seu local de origem.

**Resultados:**

As infecções mais observadas foram as do trato urinário (correlacionadas com o uso de prednisona − p<0,0001, e ciclofosfamida − p=0,045), infecções das vias aéreas superiores (correlacionadas com o uso de prednisona − p=0,0004, micofenolato de mofetila − p=0,0005 e ciclosporina − p=0,025) e pneumonia (correlacionadas ao uso de prednisona − p=0,017).

**Conclusão:**

A prednisona foi o medicamento mais associado com a presença de infecções, apontando para a necessidade de um manejo mais judicioso desse medicamento.

## INTRODUÇÃO

O lúpus eritematoso sistêmico (LES) é uma doença inflamatória crônica multissistêmica, de característica autoimune e de etiologia desconhecida, relacionada a fatores genéticos, ambientais e hormonais.^1^ Atinge predominantemente o sexo feminino em idade fértil e tende a ser mais comum e mais grave na raça negra e em asiáticos.^1^

O LES é uma doença potencialmente grave.^1^ Sendo uma vasculite de médios e de pequenos vasos, todos os órgãos estão sujeitos a danos, inclusive aqueles cuja função é vital para a sobrevivência, como coração, pulmão, rins e sistema nervoso central.^1^ No entanto, com advento do novo arsenal terapêutico e de diagnóstico precoce, as crises agudas da doença podem ser melhor controladas; a mortalidade por causa da vasculite diminuiu, e a doença assumiu um caráter mais crônico.^2)^

Em 1976, Urowitz et al.^3^ observaram um padrão bimodal nas causas de morte no LES: um pico inicial, devido à atividade da doença e infecções, e outro tardio, causado por doença arterial coronariana e infecções. Nos anos subsequentes, vários outros autores confirmaram esse padrão, que ficou sendo conhecido como curva bimodal de Urowitz.^2,3^ As infecções são uma das principais causas de morbimortalidade nessa doença, sejam elas precoces ou tardias, e todos os esforços devem ser dirigidos para sua prevenção e tratamento.

Pacientes com LES parecem possuir um aumento no risco para infecções. Entre os defeitos do sistema imune, incluem-se a redução de linfócitos T CD4+, a deficiência de componentes do sistema complemento, a neutropenia e a linfopenia.^4-6^ Alguns autores descreveram que a atividade da doença é um fator de risco para infecções, principalmente quando ela inclui o envolvimento de órgãos, como rim, e/ou de sistema nervoso central.^5,7,8^

O risco de infecções no LES é maior pelo uso de imunossupressores e particularmente pelos glicocorticoides, largamente utilizados para tratamento das complicações sistêmicas da doença.^9^ Essa última medicação ainda não possui a especificidade desejada, que seria a de atuar somente sobre células autorreativas e disfuncionais, e acaba por minar as defesas normais do organismo, favorecendo a agressão por microrganismos.

## OBJETIVO

Determinar a incidência de infecções em uma população com lúpus eritematoso sistêmico e a caracterização das mesmas quanto aos locais de origem, assim como estudar possíveis associações das infecções com a terapêutica utilizada.

## MÉTODOS

O estudo tem desenho retrospectivo analítico e utilizou dados contidos em prontuários de pacientes portadores de LES do Serviço de Reumatologia do Hospital Evangélico de Curitiba, no Paraná (PR). Após aprovação do Comitê de Ética em Pesquisa da instituição sob número 32371614.6.0000.0103, foram investigados 144 pacientes com diagnóstico de LES, que foram acompanhados pelo período de 5 anos (1^o^ de dezembro de 2007 até 1^o^ de dezembro de 2012). Foram estudados pacientes de ambos os sexos, que apresentavam, no mínimo, quatro dos critérios classificatórios para LES de acordo, com aqueles adotados pelo *American College of Rheumatology* (ACR) em 1997.^10^ Excluíram-se pacientes com idade de doença anterior aos 16 anos, indivíduos com HIV ou formas de agamaglobulinemia.

Dentre os dados coletados, incluíram-se: idade do paciente e idade de início do LES; sexo e etnia; tempo de duração da doença anterior ao período de inclusão no estudo; medicamentos utilizados; dose cumulativa de prednisona administrada; perfil clínico (avaliado de maneira cumulativa e cuja definição seguiu os critérios do ACR);^10^ e perfil sorológico, ocorrência de infecções e seu local. As infecções foram expressas em número de infecções para cada cem pacientes/ano.

Na análise estatística, as medidas de tendência central foram apresentadas como médias e desvio padrão, para amostras com distribuição gaussiana, e medianas e intervalo interquartis (IIQ), para as amostras não gaussianas. Para estudo de frequência de sintomas e achados sorológicos e de uso de medicamentos, utilizou-se a porcentagem. A correlação entre presença de infecções com medicamentos e dados epidemiológicos foi feita pelo teste de Spearman. A significância adotada foi de 5%. Os cálculos foram feitos com auxílio do pacote estatístico *Graph Pad Prism* versão 5.0.

## RESULTADOS

Dentre os pacientes estudados, 135/144 (93,8%) eram mulheres, a idade variou entre 17 a 67 anos (média de 39,15±11,65 anos) e o período de duração de doença antes da inclusão no presente estudo foi de 9 a 483 meses (mediana de 87,0; IIQ de 60,0-132,0 meses).

Os achados clínicos e sorológicos da população estudada, obtidos de maneira cumulativa durante o curso da doença, mostraram que existiam fotossensibilidade em 70,7%, eritema malar em 48,9%, úlceras orais em 45,6%, psicose em 8,3%, convulsões em 11,1%, serosite em 20,1%, leucopenia em 27,8%, plaquetopenia em 22,3%, anemia hemolítica em 4,8%, glomerulonefrites em 42,3%; anti-dsDna em 33,3%; anti-Ro em 37,3%; anti-La em 21,2%; anti-Sm em 23,5% e anti-RNP em 27,3%.

No que se refere ao uso de medicamentos, 93,7% faziam uso de antimaláricos, 31,9% de ciclofosfamida, 17,3% de micofenolato de mofetila, 31,2% de metotrexato, 6,25% de talidomida, 4,1% de ciclosporina e 86,1% de prednisona. A dose cumulativa de prednisona durante os 5 anos de observação variou de 0-56.758mg (mediana de 963mg; IIQ de 2.491-18.647mg) e foi calculada multiplicando-se a dose diária utilizada pelo número de dias de uso.

Na amostra estudada, observou-se a ocorrência de 1,68 infecção do trato urinário/100 pacientes ao ano; 1,56 infecção de vias aéreas superiores/100 pacientes ao ano; 0,47 pneumonia/100 pacientes ao ano; 0,27 herpes zóster/100 pacientes ao ano; 0,27 caso de candidíase/100 pacientes ao ano e de 0,05 caso de tuberculose/100 pacientes ao ano.

O estudo de correlação do número de infecções com os medicamentos utilizados encontra-se na tabela 1.

**Tabela 1:** Correlação entre uso de medicamentos e infecções em pacientes com lúpus eritematosos sistêmico*

Medicamento	Rho Spearman	IC95%	Valor de p
Infecções de trato urinário			
Ciclofosfamida	0,16	0,001-0,32	0,04
Prednisona (DC)	0,34	0,18-0,48	<0,0001
Infecções de vias aéreas superiores			
Micofenolato de mofetila	0,28	0,14-0,42	0,0005
Prednisona (DC)	0,28	0,12-0,43	0,0004
Ciclosporina	0,18	0,018-0,34	0,025
Pneumonia			
Prednisona (DC)	0,19	0,02-0,35	0,017
Herpes zóster			
Ciclofosfamida	0,31	0,15-0,45	0,0001
Prednisona (DC)	0,29	0,13-0,44	0,0003
Azatioprina	0,19	0,019-0,35	0,025

*As correlações de infecções com medicamentos usados pelos pacientes e que não constam no presente tabela são todas não significativas (p=NS); IC95% intervalo de confiança de 95%; DC: dose cumulativa.

A presença de tuberculose e infecções bacterianas de pele não se associaram significativamente com o uso de nenhum tipo de medicação.

## DISCUSSÃO

O LES pode ser uma doença de difícil controle.^1^ Os objetivos de seu tratamento são prevenção de surtos agudos de vasculite, evitar a lesão permanente dos órgãos afetados, manter a doença em remissão e/ou deter a sua progressão.^1^

Drogas como anti-inflamatórios não esteroidais e antimaláricos são amplamente utilizadas como tratamento. Esses são efetivos na prevenção de artrite e de manifestações cutâneas, além de, no caso dos antimaláricos, ajudar a manter a doença em remissão.^1^

Os glicocorticoides são usados especialmente no início de um surto agudo da doença, devido à sua rapidez de ação e em doses que variam de acordo com o grau e o tipo de órgão acometido.^1,10^ Apesar de mais de 60 anos de experiência em seu uso, não há diretrizes sobre a posologia ideal deste medicamento nas diferentes manifestações da doença,^11^ sendo tais doses escolhidas de acordo com o julgamento clínico da situação. A ação rápida e potente dos glicocorticoides pode, muitas vezes, salvar a vida de um paciente; mas seu uso traz uma miríade de efeitos colaterais, incluindo-se neles a facilitação para a ocorrência de infecções.^11^ Sisó et al.^12^ consideram que os glicocorticoides, junto da ciclofosfamida, são as drogas que mais favorecem infecções neste contexto. Eles têm efeitos inibidores gerais sobre as respostas imunes mediadas por linfócitos T e B, assim como potentes efeitos supressores sobre as funções de monócitos e neutrófilos, favorecendo infecções oportunistas, muitas vezes graves, como a doença invasiva pneumocócica.^13,14^ O uso de glicocorticoides tem sido associado com o aparecimento de infecções de trato respiratório e geniturinário causadas por microrganismos *Gram*-negativos^4,15^ e infecções por micobactérias, criptococos, listeria e nocardia.^16^ Um estudo retrospectivo com 2.717 pacientes com LES comparou a população que usava glicocorticoide (n=989) com a que não o utilizava (n=1.728). Esse estudo mostrou que os pacientes em uso deste medicamento numa dose média diária acima de 7,5mg/prednisona são mais suscetíveis ao desenvolvimento de pneumonia, herpes zóster e infecção fúngica.^17^ Outros autores observaram que este mesmo limiar de dose diária pode aumentar a possibilidade de infecções de trato urinário.^14,18^ Gladman et al.^19)^ postulam que o uso contínuo de corticoides – independentemente da dose, duração e via de administração, leva a um maior risco de infecções. Uma alternativa para resolver este problema seria ajustar a dose de glicocorticoide para o tipo de célula predominantemente responsável pela respectiva manifestação do LES, conhecendo-se a atuação dos glicocorticoides em diferentes tipos de células.^13^ No entanto, este conhecimento sobre a fisiopatologia da doença e mesmo sobre o modo de ação dos glicocorticoides ainda está longe de ser incorporado na prática diária.^13^ No presente estudo, os resultados encontrados foram compatíveis com a literatura, mostrando que o uso de prednisona foi associado com maior suscetibilidade para infecções de trato urinário, infecções de vias aéreas superiores e pneumonia.

Outras drogas que compõem o arsenal terapêutico no LES são os imunossupressores, como metotrexato (indicados no tratamento de manifestações como miosite, artrites e lesões cutâneas) e azatioprina (usada nas manifestações hematológicas e na manutenção de remissão de uma nefrite).^1^ Esses dois medicamentos podem ser recomendados também como poupadores de corticoides.^1^ Já a ciclofosfamida e o micofenolato de mofetila são usados nas manifestações mais graves da doença, como no acometimento de rim, sistema nervoso e vasculites maiores.^1^

A ciclofosfamida é um agente alquilante do DNA, que reduz o número de linfócitos B e T, além de modular a ativação dessas células e a produção de anticorpos.^20^ Apesar de controverso, existem autores^7,21^ que afirmam que a terapia isolada com ciclofosfamida parece não constituir um fator de risco significativo para infecções, mas que esse risco é aumentado quando a mesma é combinada com os corticoides. Na literatura, a ciclofosfamida tem sido associada com infecções de trato urinário,^21-23^ concordando com os achados do presente estudo. A ocorrência de cistite hemorrágica, uma das conhecidas complicações do uso da ciclofosfamida, pode promover o aparecimento de refluxo urinário e este, por sua vez, favorece o aparecimento de infecções.^22,23^

Um estudo retrospectivo sobre o uso do micofenolato de mofetila no LES mostrou que cistite, infecção do trato respiratório superior, bronquite e celulite foram as infecções mais frequentemente associadas com esse medicamento.^24^ Em nossa casuística, observou-se associação do uso dessa droga com infecções de vias aéreas superiores.

No presente estudo, as infecções por herpes zóster foram associadas com uso de ciclofosfamida, prednisona, azatioprina e também com o maior tempo da doença. A infecção pelo herpes zóster se deve à reativação do vírus varicela-zoster, o que pode ocorrer décadas após a exposição inicial. Alguns autores descreveram maior frequência de reativação dessa doença em pacientes lúpicos.^25,26^ Borba et al.^26^ relataram episódios de 55 infecções por herpes zóster em 51 pacientes com LES, sendo a característica mais notável deste estudo o fato de que a maioria dos episódios aconteceu após 5 anos do diagnóstico de LES, aparecendo, muitas vezes, durante períodos de remissão do mesmo.

O LES é uma doença complexa. A evolução desses pacientes é altamente variável e exige uma vigilância cuidadosa por parte do médico assistente. O tratamento, embora tenha grande impacto sobre a sobrevida e a qualidade de vida dessa população, pode predispor a infecções repetidas e, eventualmente, graves. Cabe ao médico que acompanha este paciente o uso judicioso deste arsenal terapêutico, principalmente dos glicocorticoides, utilizando-os pelo menor tempo e na menor dose possível.

## CONCLUSÃO

A infecção mais comum nesta amostra de pacientes com lúpus eritematoso sistêmico foi a do trato urinário, seguida pela de vias aéreas superiores. As infecções do trato urinário foram significativamente associadas com uso de prednisona e ciclofosfamida; já as do trato respiratório, com uso de prednisona, de micofenolato de mofetila e de ciclosporina.

De forma geral, o glicocorticoide foi o medicamento que mais apresentou associação com a presença de infecções, apontando para a necessidade de um manejo adequado desse medicamento.
